# Alternative Performance Measures for Prediction Models

**DOI:** 10.1371/journal.pone.0091249

**Published:** 2014-03-07

**Authors:** Yun-Chun Wu, Wen-Chung Lee

**Affiliations:** 1 Institute of Epidemiology and Preventive Medicine, College of Public Health, National Taiwan University, Taipei, Taiwan; 2 Research Center for Genes, Environment and Human Health, College of Public Health, National Taiwan University, Taipei, Taiwan; University of Texas MD Anderson Cancer Center, United States of America

## Abstract

As a performance measure for a prediction model, the area under the receiver operating characteristic curve (AUC) is insensitive to the addition of strong markers. A number of measures sensitive to performance change have recently been proposed; however, these relative-performance measures may lead to self-contradictory conclusions. This paper examines alternative performance measures for prediction models: the Lorenz curve-based Gini and Pietra indices, and a standardized version of the Brier score, the scaled Brier. Computer simulations are performed in order to study the sensitivity of these measures to performance change when a new marker is added to a baseline model. When the discrimination power of the added marker is concentrated in the gray zone of the baseline model, the AUC and the Gini show minimal performance improvements. The Pietra and the scaled Brier show more significant improvements in the same situation, comparatively. The Pietra and the scaled Brier indices are therefore recommended for prediction model performance measurement, in light of their ease of interpretation, clinical relevance and sensitivity to gray-zone resolving markers.

## Introduction

Risk prediction models are important for both patients and physicians alike. A prediction model can be used to integrate an individual’s socio-demographic variables, medical histories and biomarker values, etc., and to translate them into a disease risk, upon which prognostication and/or treatment decision can be based. Examples are the prediction models for cardiovascular diseases [1], hypertension [2], diabetes [3] and different forms of cancer [4]–[6]. Prediction model performance must be evaluated in a scientific way. There are two aspects to model performance: calibration and discrimination. Calibration is a measure of how well predicted probability agrees with actual observed risk, while discrimination is a measure of how well a model separates those who do and do not have the disease of interest [7]. This study focuses on evaluating the discrimination ability of a prediction model.

The area under the receiver operating characteristic (ROC) curve (AUC) (also referred to as the c statistic) is by far the most popular index of discrimination ability [8]. AUC is defined as the probability that the predicted probability of a randomly selected diseased subject will exceed that of a randomly selected non-diseased subject. AUC is a value between 0.5 and 1.0, with a higher value indicating better prediction performance. A prediction model with an AUC value of 0.5 is no better than tossing a coin, and at the other extreme, a model with a 1.0 AUC value is a perfect model, with 100% accurate predictions. However, AUC has been criticized as insensitive to the addition of strong marker(s), typically resulting in only small changes in value [9], [10]. A small change in AUC (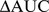), even though it is statistically significant, can be difficult to interpret. For example, the addition of C-reactive protein to a set of standard risk factors predicting cardiovascular disease only increases the model AUC from 0.72 to 0.74 [11], and the 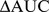 is a mere 0.001 (from 0.900 to 0.901) when a genotype score (derived from a total of 18 alleles) is added into the prediction model for type 2 diabetes [3]. One cannot help wondering whether this is because the C-reactive protein and the genotype score (despite their strong associations with the disease) are actually useless in disease prediction, or whether the AUC’s insensitivity to model performance change is entirely to blame.

Recently, a number of ‘relative-performance’ indices that are sensitive to performance change have been proposed [12]. These measures specifically compare models with and without new markers, and include net reclassification improvement (NRI), continuous NRI (cNRI) and integrated discrimination improvement (IDI) [13], [14]. NRI is defined as the difference between the proportion of subjects ‘moving up’ (changing to higher risk categories in the model with the new marker(s)) and the proportion of subjects ‘moving down’ (changing to lower risk categories) for diseased subjects, and the corresponding difference in proportions for non-diseased subjects [13]. cNRI and IDI also hinge on such up and down movement. In cNRI, any increase (decrease) in predicted probability constitutes a movement up (down) [14]. In IDI, the actual amount of increase/decrease in predicted probability is counted [13]. However, a relative-performance measure can sometimes lead to self-contradictory conclusions. For example, a situation may occur in which the prediction performances of models A, B and C are rated, using a relative performance index, as A>B and B>C, yet paradoxically, A<C.

This paper describes and compares a number of alternative performance measures for prediction models. These include the Lorenz curve-based Gini and Pietra indices [15] and a standardized version of the Brier score, the scaled Brier (sBrier) [7]. All these are absolute measures, directly reflecting the prediction performance of a specific model, and when used for model comparisons they do not produce self-contradictory results. The sensitivity of these measures to performance change when new marker(s) are added to a baseline model will also be examined.

## Methods

### Formulas for Various Performance Measures

**Figure 1:**
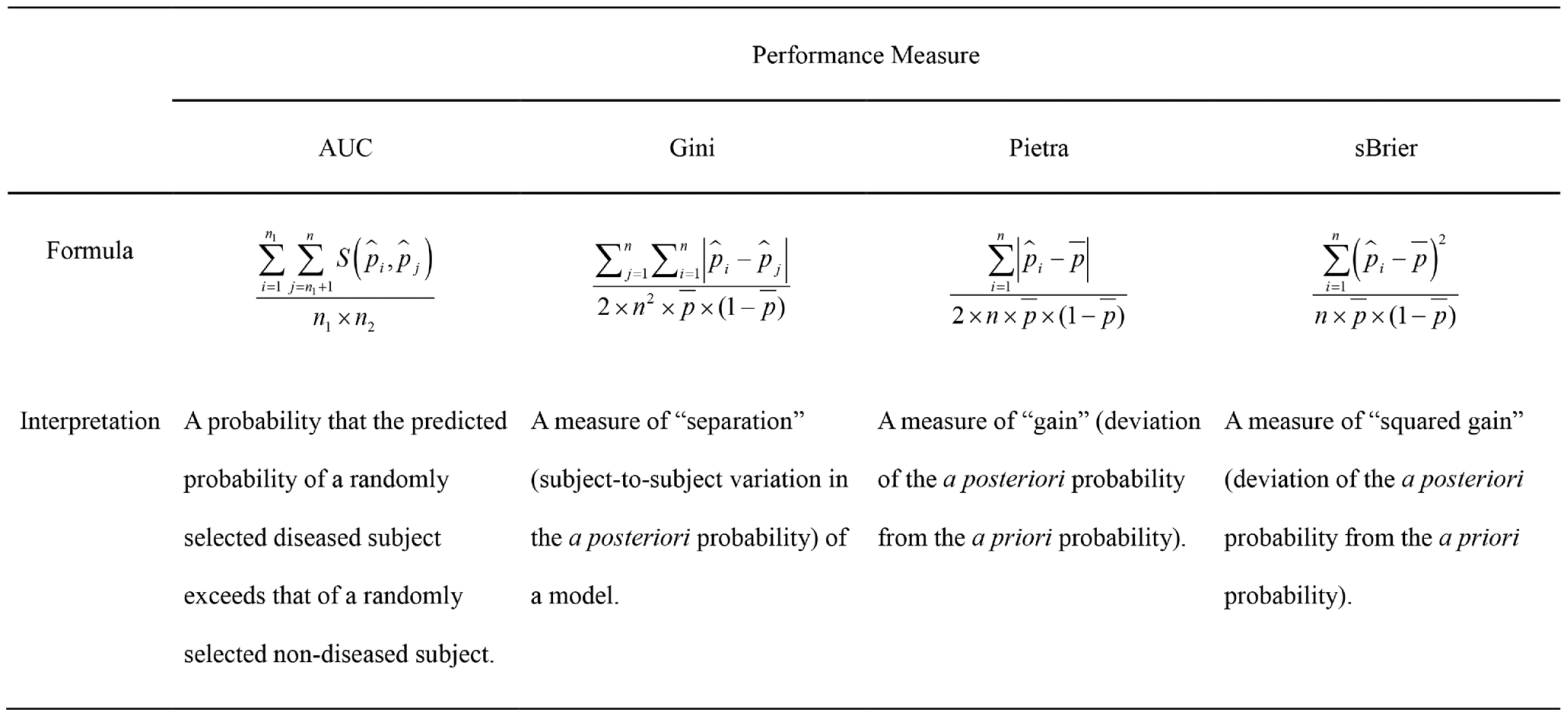
Computing formulas and interpretations of various performance measures.

Assume that there are a total of 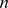 subjects (indexed 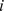) in a population, of which 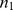 (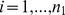) subjects are diseased (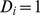), and 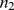 (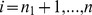) subjects are non-diseased (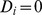). Assume a prediction model which yields a predicted probability, 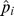, for each and every subject in the population. The prediction model is well calibrated and unbiased such that the mean predicted probability, 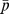, is equal to disease prevalence in the population, that is, 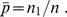 Figure 1 presents the computing formulas and interpretations of various performance measures, including AUC, Gini, Pietra and sBrier.

The formula for AUC is
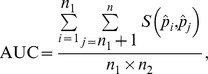
where 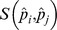 is a scoring function comparing the predicted probabilities for a pair of subjects: 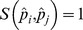 if 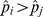, 0.5 if 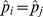, and 0 if otherwise. The formula clearly shows that AUC is the probability that the predicted probability of a randomly selected diseased subject exceeds that of a randomly selected non-diseased subject.

It is of interest to compare the computing formulas for Gini, Pietra and sBrier:
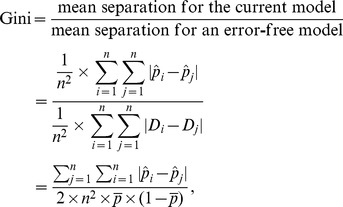





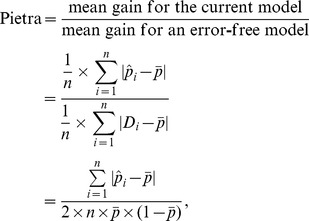
and



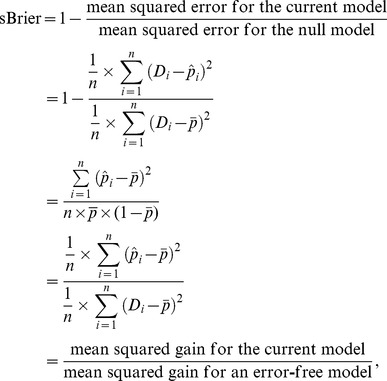
respectively. Note that initially, all subjects in the population are on the same footing - the same *a priori* probability (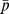). When a prediction model is used, however, they diverge (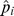
s are different in general). Gini quantifies the “separation” (subject-to-subject variation in the *a posteriori* probability) of a model, while Pietra and sBrier quantify the “gain” (deviation of the *a posteriori* probability from the *a priori* probability).

### Simulation Schemes

Three variables are assumed to be predictive of a particular disease (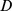): the baseline score (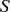) and two new markers (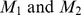). It is assumed that 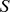 is a composite of traditional risk factors (age, smoking, systolic blood pressure, total and high density lipoprotein cholesterol levels, etc.) standardized to a normal distribution with a mean of 0 and a standard deviation of 1. The new markers are assumed to be binary. In order to acknowledge a correlation between 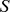 and the two new markers, let the prevalence of 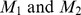 be 85% when 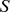 is above average (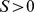), and 75%, when otherwise.

**Figure 2:**
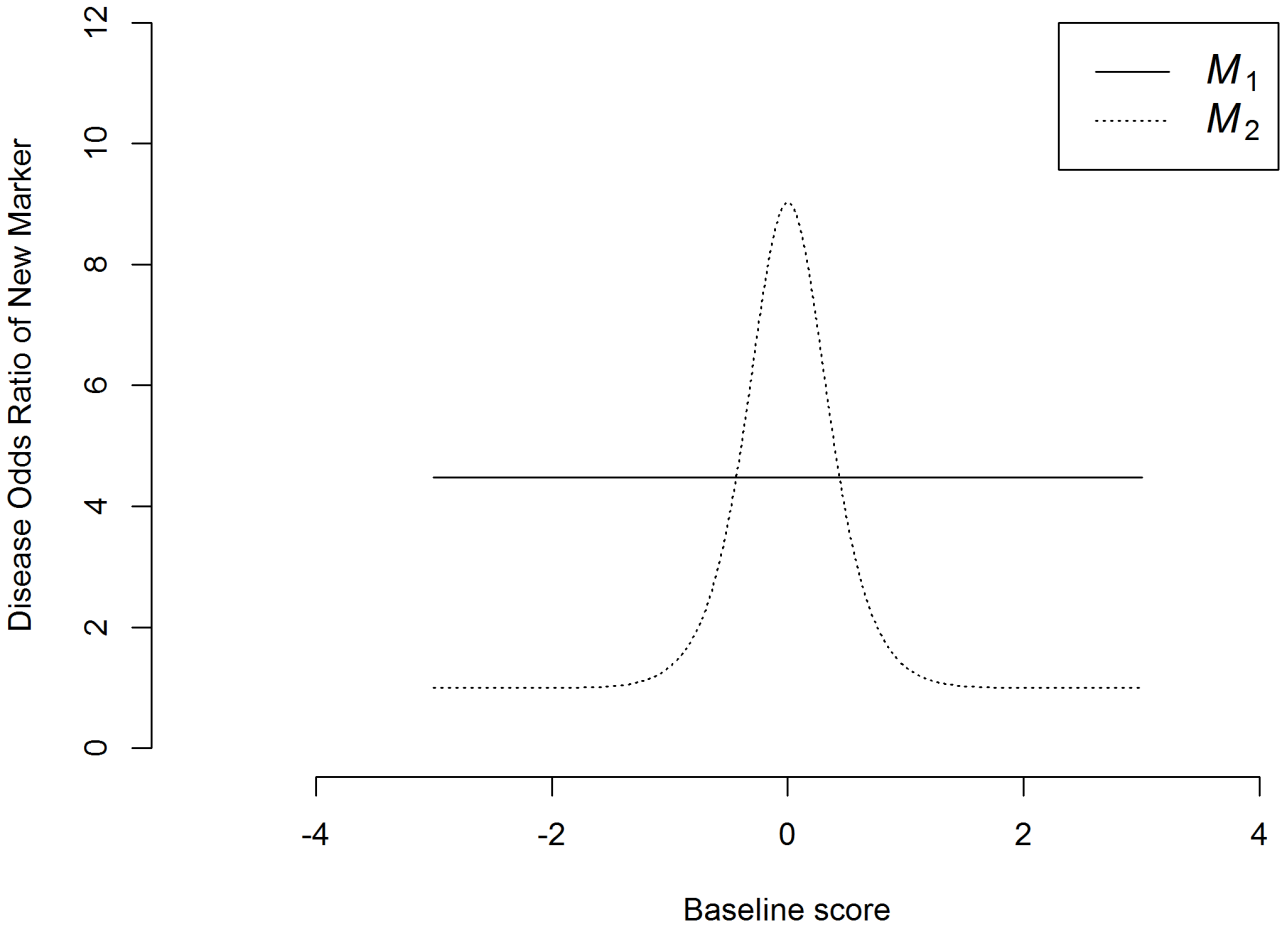
Disease odds ratios (discrimination powers) of the new markers (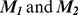) (solid line: when the discrimination power of the new marker (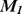) is independent of the baseline score; dotted line: when the discrimination power of the new marker (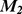) is concentrated in the gray zone of the baseline model).

It is assumed that the discrimination power of 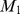 is independent of the baseline score, whereas the discrimination power of 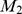 is not uniform, but is concentrated in the gray zone of the baseline model (where the predicted probability using the baseline model is close to the *a priori* probability). Specifically, the disease risk is assumed to follow a logistic model, as below:
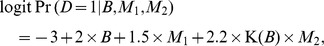
where 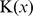 is a Gaussian kernel function centered at 0: 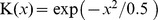. In this model, the disease odds ratio per unit increase in the baseline score (disease odds ratio for one standard deviation increase in the composite variable of traditional risk factors) is 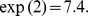 To simulate new markers that are strong predictors for the disease, we let the disease odds ratio for 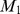 to be 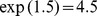 irrespective of the baseline score (Figure 2), and the disease odds ratio for 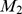 to reach a peak [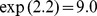] when the baseline score is at its average value (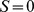) and rapidly decay when the baseline score is above or below average (Figure 2).

A total of 500 subjects were simulated as the training sample, and another 500 subjects were simulated as the validation sample. The performances of three prediction models were compared: (I) the model with the baseline score only, (II) the model with the baseline score plus 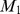 and (III) the model with the baseline score plus 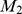. A total of 10000 simulations were performed.

## Results

**Figure 3:**
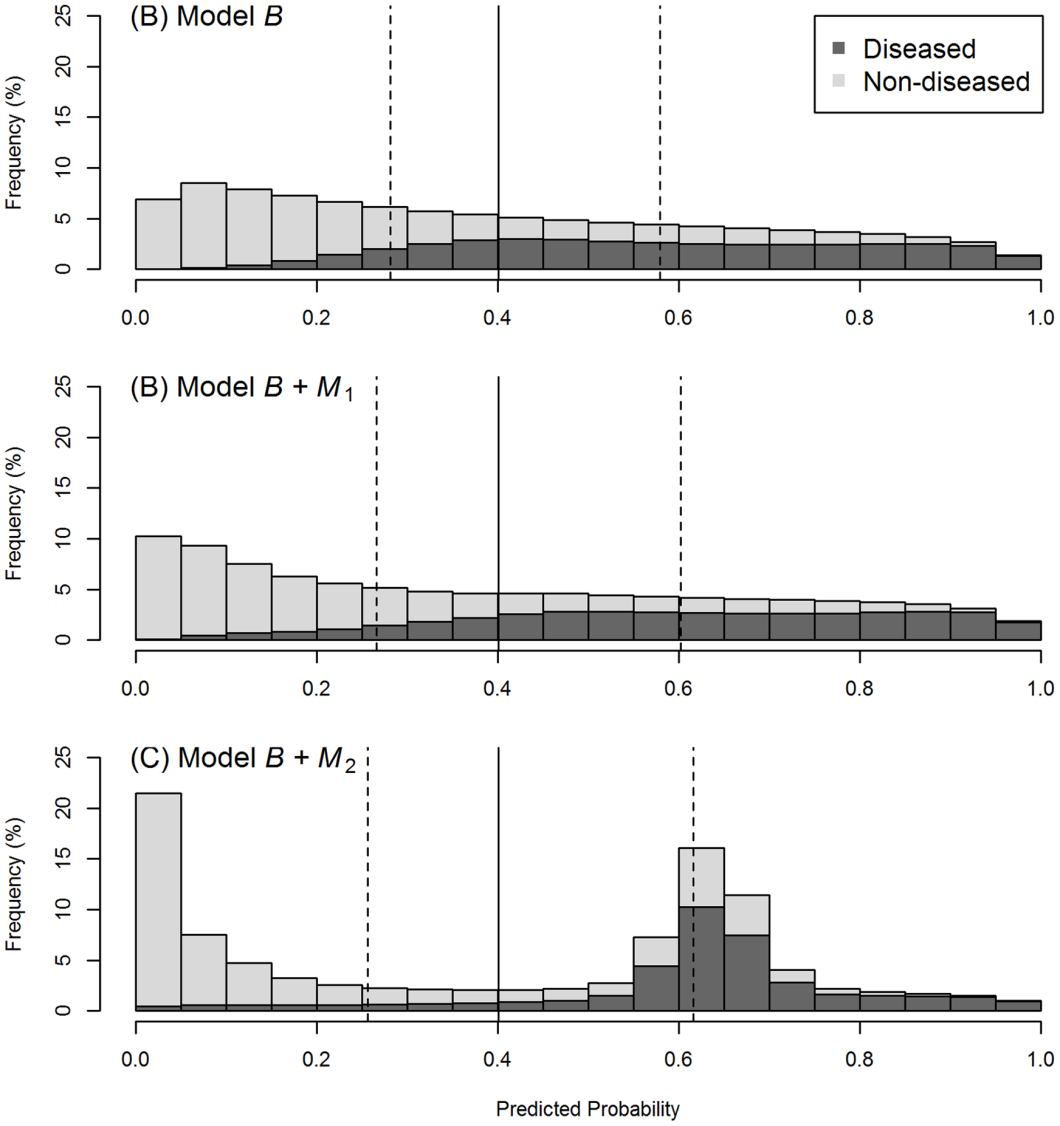
Distribution of the predicted probabilities for a baseline model (A), and the model with the new marker 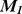 added (B), or 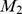 added (C). The discrimination power of 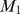 is independent of the baseline score, and that of 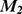 is concentrated in the gray zone of the baseline model. The solid vertical bar indicates the grand mean of the predicted probabilities, and the two dotted vertical bars, the means of the predicted probabilities for the diseased subjects and the non-diseased subjects, respectively.

In Figure 3, it can be seen that there is almost no change in the distributions of the predicted probabilities between the baseline model (A) and the model with 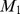 added (B). Using the AUC index, it can be seen that adding 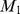 increases the prediction performance of the model from 0.822 to 0.841, an absolute (relative) improvement of a mere +0.019 (+2.3%) (Table 1). Noted that the absolute improvement gauged by the Gini index (+0.039) is twice that by AUC (apart from the rounding error; in fact, 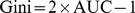, see [15]), and the relative improvement is +6.1%. The Pietra [+0.036 (+7.4%)] and the sBrier [+0.038 (+12.4%)] also demonstrate more significant improvements than that of AUC.

**Table 1:** Improvements in prediction performances when new markers, 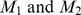, are added to a baseline model (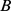), respectively.

	Performance Measure			
	AUC	Gini	Pietra	sBrier
Model				
* B*	0.822	0.644	0.485	0.306
* B+M*_1_	0.841	0.683	0.521	0.344
* B+M*_2_	0.844	0.687	0.568	0.363
Absolute (Relative) Improvement				
from *B* to *B+M*_1_	+0.019 (+2.3%)	+0.039 (+6.1%)	+0.036 (+7.4%)	+0.038 (+12.4%)
from *B* to *B+M*_2_	+0.022 (+2.7%)	+0.043 (+6.7%)	+0.083 (+17.1%)	+0.057 (+18.6%)

The discrimination power of 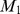 is independent of the baseline score, whereas that of 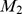 is concentrated in the gray zone of the baseline model.

By contrast, the results are much more intriguing when 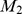 is added. In Figure 3, the number of people (diseased or non-diseased) in the gray zone (near the solid vertical bars) is drastically reduced when 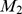 is added (C) to the baseline model (A); most diseased individuals move to the right (higher predicted probability), whereas most non-diseased individuals move to the left. An informative marker like 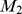 certainly deserves a high rate; however, the AUC credits it with an absolute (relative) improvement in prediction performance of only +0.022 (+2.7%), and the Gini, twice that value, but still only +0.043 (+6.7%) (Table 1). Comparatively, the Pietra [+0.083 (+17.1%)] and the sBrier [+0.057 (+18.6%)] indices more fittingly judge the value of the marker.

It is also of interest to compare models “
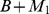
” and “
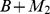
” head to head. Figure 3 shows that the two models generate predicted probabilities that are quite different in distribution (B vs. C); however, AUC and Gini fail to set them apart (AUC: 0.841 vs. 0.844; Gini: 0.683 vs. 0.687). By contrast, Pietra and sBrier clearly differentiate between the two models (Pietra: 0.521 vs. 0.568; sBrier: 0.344 vs. 0.363).

In addition, this study examined situations when a strong continuous-scale marker (Exhibit S1) and multiple weak binary markers (Exhibit S2; to simulate genetic markers that are by themselves weak predictors for the disease but are strongly predictive of the disease if used collectively as a genetic score) were added to the baseline model, respectively. The conclusions regarding the comparisons of the various performance indices remain the same as when one strong binary marker is added, as shown above.

## Discussion

ROC curve analysis is the most widely used method for the evaluation of diagnostic test or prediction model performance [16]–[19]. For any subject to be diagnosed/predicted, a diagnostic test yields a single test value which, depending on the test used, can be in binary, ordinal or continuous scale, whereas a prediction model, upon integrating the information of more than one predictor, produces a probability, which is a value between 0 and 1. Lorenz curve analysis has also enjoyed a long history of use, dating back to 1905 [20]. However, it has been primarily used by economists (demographers) to study inequality in income (population) distribution [21], [22]. Lee [15] pioneered the use of Lorenz curve analysis in biomedicine (in the context of diagnostic test evaluation, although he did not consider prediction models). The interpretation of the ROC curve-based AUC index is actually rather unrealistic - subjects will not come in pairs, one being diseased and the other non-diseased, with their predicted probabilities to be compared. By contrast, Lorenz curve-based Gini and Pietra indices follow-up study subjects from their *a priori* probabilities to their *a posteriori* probabilities (after using a prediction model), and should have more relevance for actual clinical practices.

Brier score has been used to evaluate the accuracy of weather forecasting since 1950 [23]. In recent decades it has seen use in applications in biomedical fields [24]–[26]. Brier score depends on the disease prevalence (the *a priori* probability) of the population where the prediction model is built, and therefore it is unsuitable for making a comparison between populations. Steyerberg et al. [7] proposed a standardized version of the Brier score, the sBrier, which is an index between 0 and 1, and is prevalent-independent. Austin and Steyerberg [27] used sBrier to examine performance changes when new markers were added to a baseline model. However, they did not consider the type of markers with discrimination power concentrating in the gray zone of the baseline model, and therefore did not recognize that sBrier was sensitive to gray-zone resolving markers. Another, lesser known fact about sBrier is that the change in sBrier upon addition of new markers is equal to the IDI index itself. A proof of this is given in Exhibit S3.

It is worth noting that Gini, Pietra and sBrier indices can be expressed as ratios, comparing the resolution power (separation for Gini; gain for Pietra; squared gain for sBrier) of the current model with that of an error-free model. They are all therefore indices between 0 and 1, and can be neatly interpreted as a per cent maximum resolution power of the current model. In Table 1, the prediction performances of the baseline model are 0.644 (Gini), 0.485 (Pietra), and 0.306 (sBrier), respectively. This means that the baseline model still has a great deal of room for improvement; currently, it only achieves 64.4% separation/48.5% gain/30.6% squared gain of a sure-fire prediction model.

In this study, it is felt that patients (and their physicians) should be more interested in the gain (or squared gain) of a model (this tells how much their disease probability could be expected to be revised if they use that model), than in the separation (this compares two randomly chosen people). This study found that the two indices that quantify gains (Pietra and sBrier) are also those that are most sensitive to gray-zone resolving markers.

Taken together, Pietra and sBrier are promising alternative prediction model performance measures, in light of their ease of interpretation, clinical relevance and sensitivity to gray-zone resolving markers. Further work is needed to fully develop the statistical inference procedures (hypothesis tests and confidence intervals etc.) regarding these two indices.

## Supporting Information

Exhibit S1 **Simulation when a strong continuous-scale marker is added to the prediction model.**
(PDF)

Exhibit S2 **Simulation when multiple weak binary markers are added to the prediction model.**
(PDF)

Exhibit S3 **A proof that the change in sBrier upon addition of new marker(s) is equal to the IDI index.**
(PDF)
