# Supplementary material for: Alternative Performance Measures for Prediction Models
Source: PLoS One. 2014 Mar 7;9(3):e91249. doi: 10.1371/journal.pone.0091249 (PMC3946724; doi:10.1371/journal.pone.0091249)
Supplement: Exhibit S1 — Simulation when a strong continuous-scale marker is added to the prediction model. (PDF) [file pone.0091249.s001.pdf]

Exhibit S1. Simulation when a strong continuous-scale marker is added to the prediction model.

Three variables are assumed to be predictive of a particular disease (D): the baseline score (S) and two new markers (M_3 and M_4). S is the same composite baseline variable as in the text, whereas the new markers now are assumed to be continuous (normally distributed). In order to acknowledge a correlation between S and the two new markers, let the mean of M_3 and M_4 be 3.65 and 0.05 respectively when S is above average ($S > 0$), and 3.55 and -0.05, when otherwise. The standard deviations of both M_3 and M_4 are assumed to be 1.

It is assumed that the discrimination power of M_3 is independent of the baseline score, whereas the discrimination power of M_4 is not uniform, but is concentrated in the gray zone of the baseline model (where the predicted probability using the baseline model is close to the *a priori* probability). Specifically, the disease risk is assumed to follow a logistic model, as below:

$$\text{logit } \Pr(D=1 \mid B, M_3, M_4) = -3 + 2\times B + 0.8\times M_3 + 2.2\times \mathrm{K}(B)\times M_4,$$

where $\mathrm{K}(x)$ is a Gaussian kernel function centered at 0: $\mathrm{K}(x) = \exp\left(-x^2/0.0833\right)$. In this model, the disease odds ratio per unit increase in the baseline score is $\exp(2) = 7.4$ (the same as in the text). The disease odds ratio per unit increase in M_3 is $\exp(0.8) = 2.2$ irrespective of the baseline score. The disease odds ratio per unit increase in M_4 reaches a peak [$\exp(2.2) = 9.0$] when the baseline score is at its average value ($S = 0$), and rapidly decays when the baseline score is above or below

average:

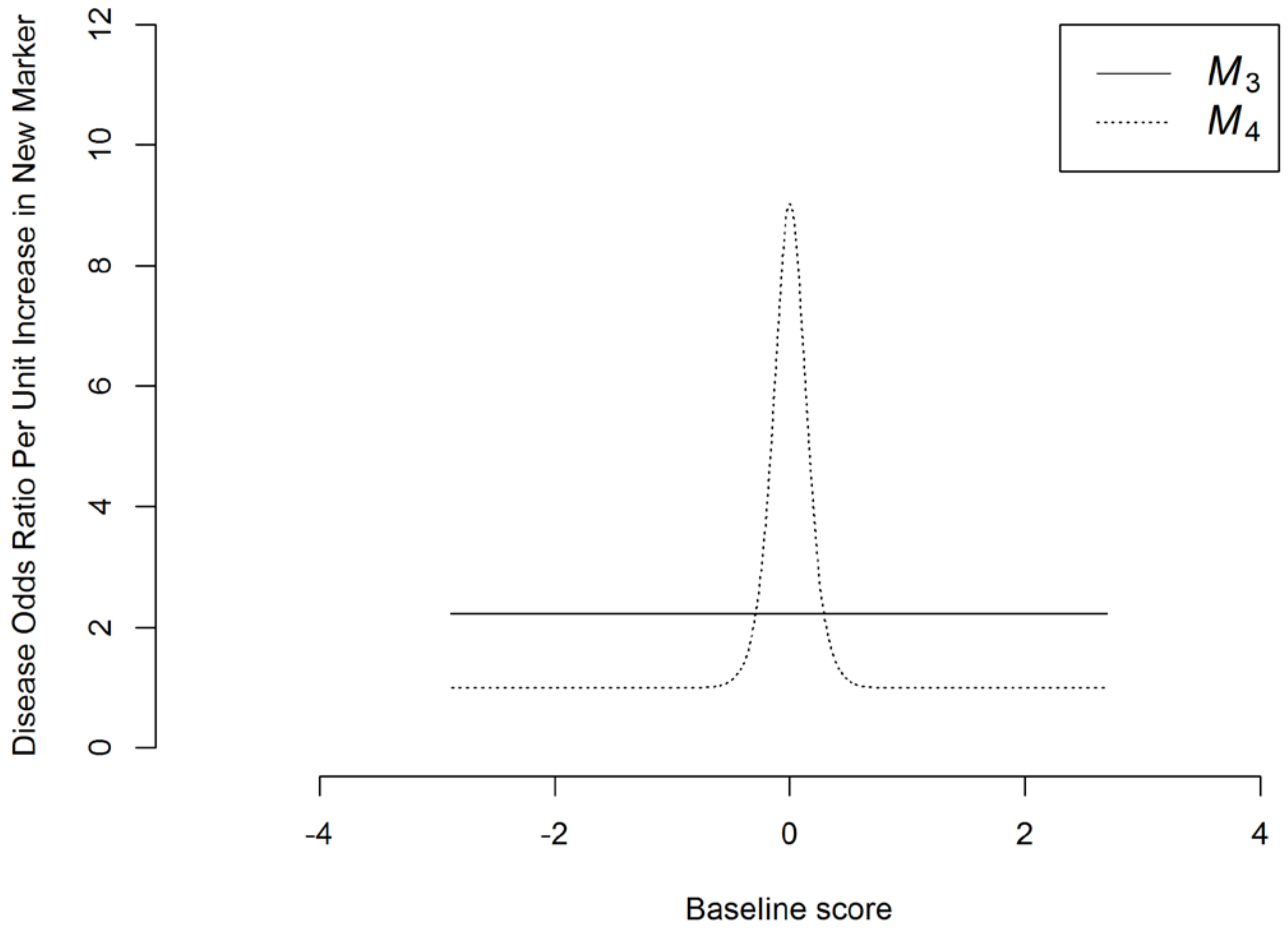

Disease Odds Ratio Per Unit Increase in New Marker
0
2
4
6
8
10
12
-4
-2
0
2
4
Baseline score
M_3
M_4

A total of 500 subjects were simulated as the training sample, and another 500 subjects were simulated as the validation sample. The performances of three prediction models were compared (see below): (I) the model with the baseline score only, (II) the model with the baseline score plus M_3 and (III) the model with the baseline score plus M_4. A total of 10000 simulations were performed.

	**Performance Measure**			
	AUC	Gini	Pietra	sBrier
**Model**				
B	0.848	0.696	0.531	0.364
$B+M_3$	0.872	0.744	0.577	0.419
$B+M_4$	0.874	0.749	0.611	0.433
**Absolute (Relative) Improvement**				
from B to $B+M_3$	+0.024 (+2.8%)	+0.048 (+6.9%)	+0.046 (+8.7%)	+0.055 (+15.1%)
from B to $B+M_4$	+0.026 (+3.1%)	+0.053 (+7.6%)	+0.080 (+15.1%)	+0.069 (+19.0%)

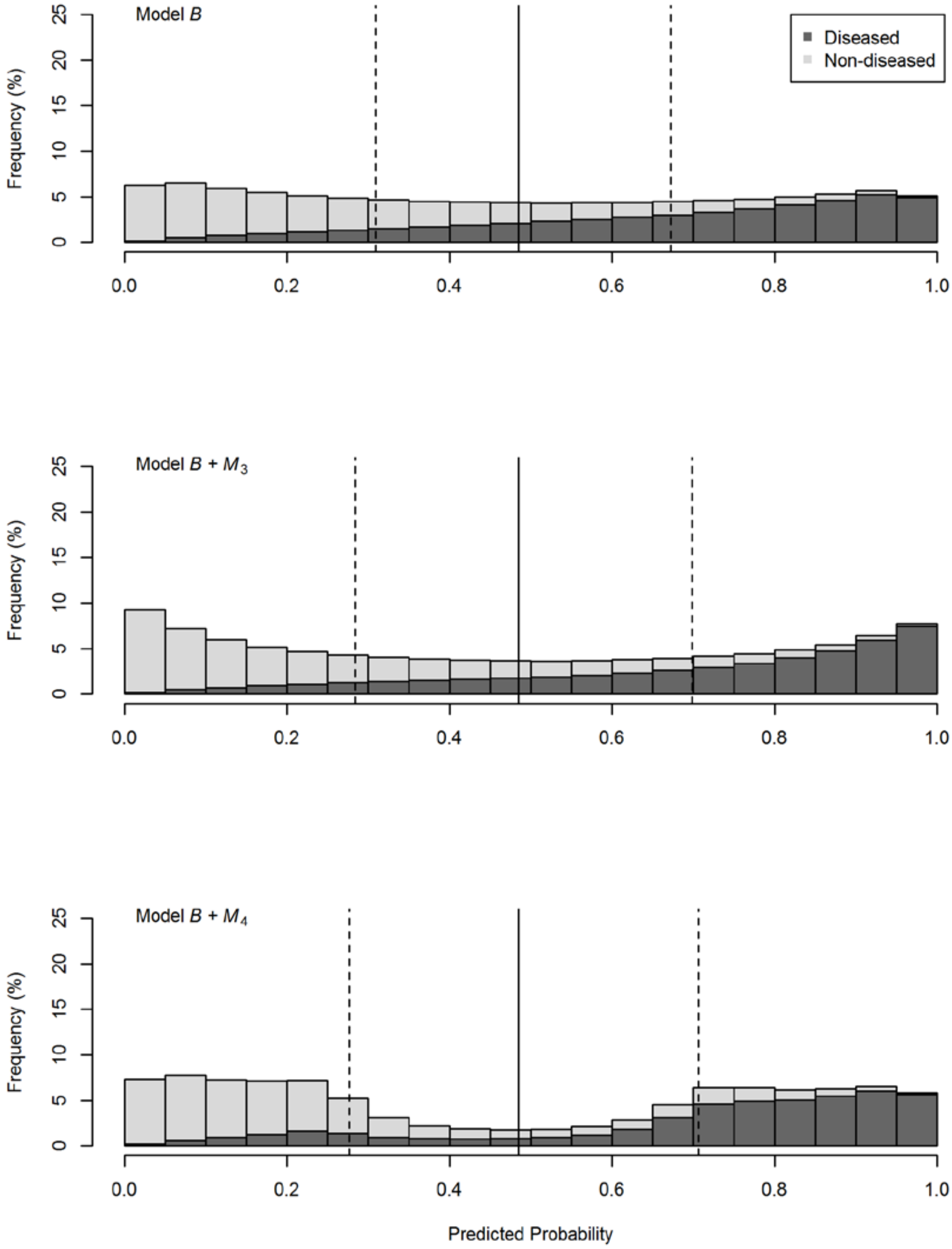
Model B
Diseased
Non-diseased
Model $B + M_3$
Model $B + M_4$
Frequency (%)
0
5
10
15
20
25
0.0
0.2
0.4
0.6
0.8
1.0
Predicted Probability
