# Supplementary material for: Alternative Performance Measures for Prediction Models
Source: PLoS One. 2014 Mar 7;9(3):e91249. doi: 10.1371/journal.pone.0091249 (PMC3946724; doi:10.1371/journal.pone.0091249)
Supplement: Exhibit S2 — Simulation when multiple weak binary markers are added to the prediction model. (PDF) [file pone.0091249.s002.pdf]

Exhibit S2. Simulation when multiple weak binary markers are added to the prediction model.

Seven variables are assumed to be predictive of a particular disease (D): the baseline score (S) and six new markers (M_5, M_6, M_7, M_8, M_9 and M_{10}). S is the same composite baseline variable as in the text. The new markers are assumed to be binary. In order to acknowledge a correlation between S and the six new markers, let the prevalence of M_5, M_6, M_7, M_8, M_9 and M_{10} be 85% when S is above average ($S > 0$), and 75%, when otherwise.

The new markers, M_5, M_6 and M_7, represent three genetic markers with additive effect. It is assumed that the discrimination power of the genetic score ($M_5 + M_6 + M_7$) is independent of the baseline score. The new markers, M_8, M_9 and M_{10}, represent another three genetic markers with additive effect. The discrimination power for the genetic score ($M_8 + M_9 + M_{10}$) is concentrated in the gray zone of the baseline model (where the predicted probability using the baseline model is close to the *a priori* probability). Specifically, the disease risk is assumed to follow a logistic model, as below:

$$\begin{aligned}\text{logit } \Pr(D=1 \mid B, M_5, M_6, M_7, M_8, M_9, M_{10}) &= -3 + 2 \times B + 0.7 \times \left(M_5 + M_6 + M_7\right) \\ &\quad + 0.75 \times \mathrm{K}(B) \times \left(M_8 + M_9 + M_{10}\right),\end{aligned}$$

where $\mathrm{K}(x)$ is a Gaussian kernel function centered at 0: $\mathrm{K}(x) = \exp\left(-x^2/0.5\right)$. In this model, the disease odds ratio per unit increase in the baseline score is $\exp(2) = 7.4$ (the same as in the text). To simulate genetic markers that are by themselves weak predictors

for the disease but are strongly predictive of the disease if used collectively as a genetic score, we let the disease odds ratio per unit increase in the genetic score ($M_5 + M_6 + M_7$) to be $\exp(0.7) = 2.0$ irrespective of the baseline score, and the disease odds ratio per unit increase in the genetic score ($M_8 + M_9 + M_{10}$) to reach a peak [$\exp(0.75) = 2.1$] when the baseline score is at its average value ($S = 0$) and rapidly decay when the baseline score is above or below average:

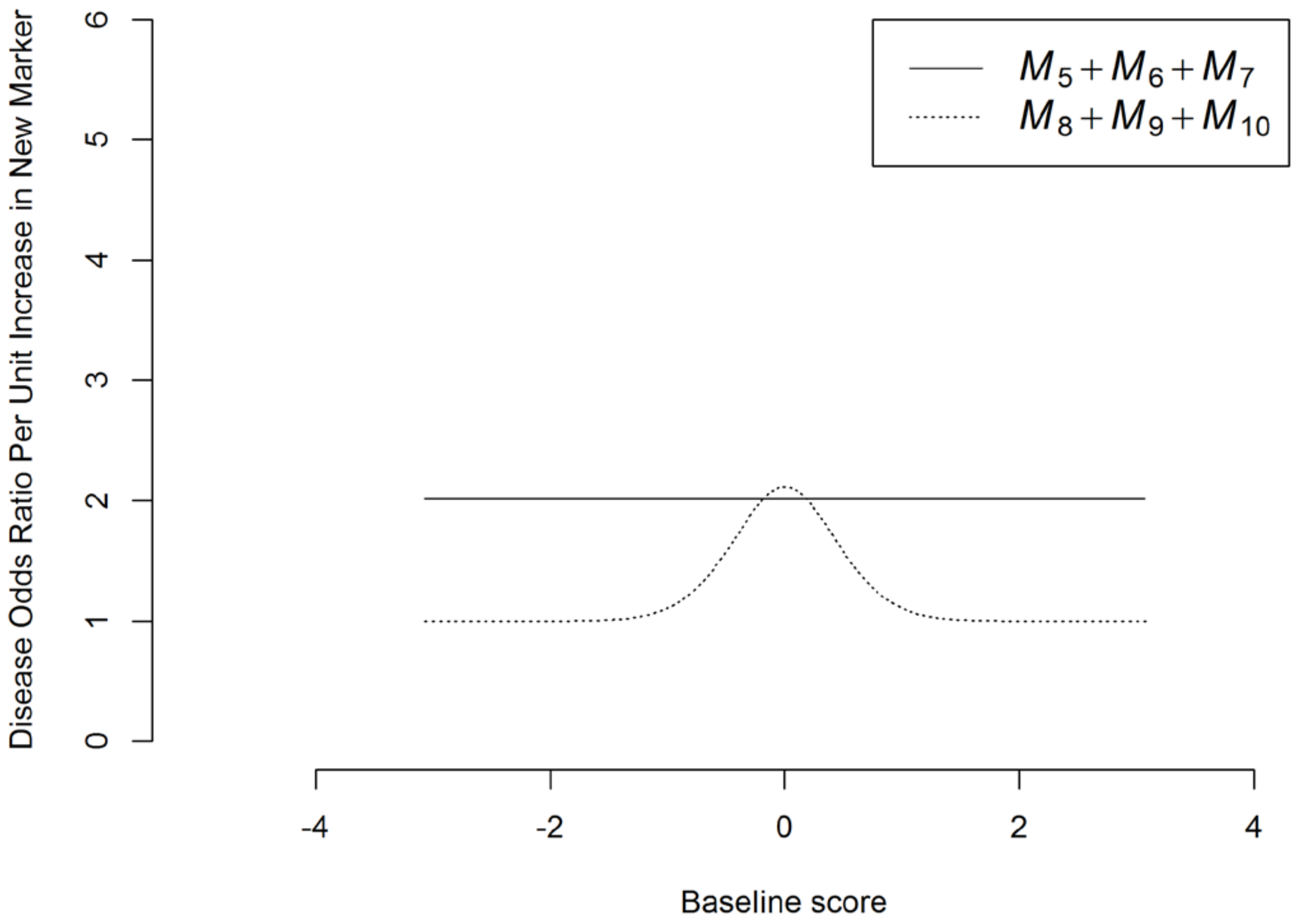

Disease Odds Ratio Per Unit Increase in New Marker
0
1
2
3
4
5
6
$M_5+M_6+M_7$
$M_8+M_9+M_{10}$
-4
-2
0
2
4
Baseline score

A total of 500 subjects were simulated as the training sample, and another 500 subjects were simulated as the validation sample. The performances of three prediction models were compared (see below): (I) the model with the baseline score only, (II) the model with the baseline score plus the genetic score, $M_5 + M_6 + M_7$, (III) the model with the baseline score plus the genetic score, $M_8 + M_9 + M_{10}$. A total of 10000 simulations were performed.

	Performance Measure			
	AUC	Gini	Pietra	sBrier
**Model**				
B	0.844	0.689	0.525	0.357
$B+M_5+M_6+M_7$	0.859	0.717	0.556	0.388
$B+M_8+M_9+M_{10}$	0.858	0.717	0.585	0.407
**Absolute (Relative) Improvement**				
from B to $B+M_5+M_6+M_7$	+0.015 (+1.8%)	+0.028 (+4.1%)	+0.031 (+5.9%)	+0.031 (+8.7%)
from B to $B+M_8+M_9+M_{10}$	+0.014 (+1.7%)	+0.028 (+4.1%)	+0.060 (+11.4%)	+0.050 (+14.0%)

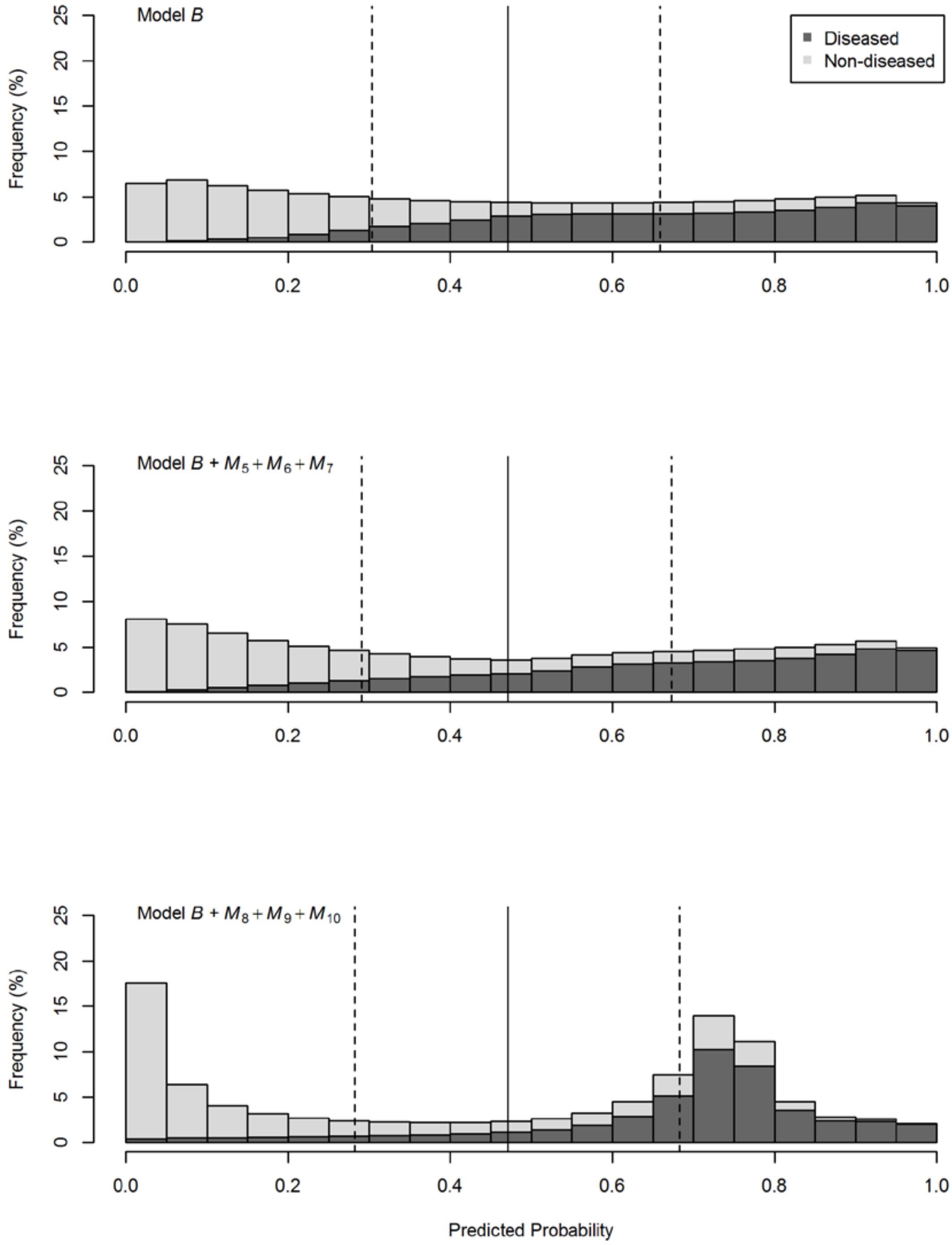

Model B
Diseased
Non-diseased
Frequency (%)
0
5
10
15
20
25
0.0
0.2
0.4
0.6
0.8
1.0
Model $B + M_5 + M_6 + M_7$
Frequency (%)
Model $B + M_8 + M_9 + M_{10}$
Frequency (%)
Predicted Probability
