# Supplementary material for: Alternative Performance Measures for Prediction Models
Source: PLoS One. 2014 Mar 7;9(3):e91249. doi: 10.1371/journal.pone.0091249 (PMC3946724; doi:10.1371/journal.pone.0091249)
Supplement: Exhibit S3 — A proof that the change in sBrier upon addition of new marker(s) is equal to the IDI index. (PDF) [file pone.0091249.s003.pdf]

Exhibit S3. A proof that the change in sBrier upon addition of new marker(s) is equal to the IDI index.

We use a star in superscript to denote the prediction model with the new marker(s) added. First, we note that the prediction models with and without the new marker(s) are to be applied to the same population and that both models are unbiased. Therefore, $\overline{p}^* = \overline{p}$. With simple algebra, we next show that the change in sBrier upon addition of new marker(s) is equal to the IDI index:

$$
\begin{aligned}
\Delta\text{sBrier} &= \text{sBrier}^* - \text{sBrier} \\
&= \frac{\sum_{i=1}^{n}\left(\hat{p}_i^* - \overline{p}^*\right)^2}{n \times \overline{p}^* \times (1-\overline{p}^*)} - \frac{\sum_{i=1}^{n}\left(\hat{p}_i - \overline{p}\right)^2}{n \times \overline{p} \times (1-\overline{p})} \\
&= \frac{\left(\sum_{i=1}^{n}\hat{p}_i^{*2} - n \times \overline{p}^{*2}\right) - \left(\sum_{i=1}^{n}\hat{p}_i^{2} - n \times \overline{p}^{2}\right)}{n \times \overline{p} \times (1-\overline{p})} \\
&= \frac{\left[\sum_{i=1}^{n_1}\hat{p}_i^* - \overline{p} \times \left(\sum_{i=1}^{n}\hat{p}_i^*\right)\right] - \left[\sum_{i=1}^{n_1}\hat{p}_i - \overline{p} \times \left(\sum_{i=1}^{n}\hat{p}_i\right)\right]}{n \times \overline{p} \times (1-\overline{p})} \\
&= \frac{\left[\left(1-\overline{p}\right) \times \sum_{i=1}^{n_1}\hat{p}_i^* - \overline{p} \times \sum_{j=n_1+1}^{n}\hat{p}_j^*\right] - \left[\left(1-\overline{p}\right) \times \sum_{i=1}^{n_1}\hat{p}_i - \overline{p} \times \sum_{j=n_1+1}^{n}\hat{p}_j\right]}{n \times \overline{p} \times (1-\overline{p})} \\
&= \frac{\sum_{i=1}^{n_1}\hat{p}_i^* - \sum_{i=1}^{n_1}\hat{p}_i}{n \times \overline{p}} - \frac{\sum_{j=n_1+1}^{n}\hat{p}_j^* - \sum_{j=n_1+1}^{n}\hat{p}_j}{n \times (1-\overline{p})} \\
&= \frac{\sum_{i=1}^{n_1}\left(\hat{p}_i^* - \hat{p}_i\right)}{n_1} - \frac{\sum_{j=n_1+1}^{n}\left(\hat{p}_j^* - \hat{p}_j\right)}{n_2} \\
&= \text{IDI.}
\end{aligned}
$$
